# Are Coping Strategies with Well-Being in Deaf and Blind Parents Related?

**DOI:** 10.3390/ejihpe11040102

**Published:** 2021-11-17

**Authors:** Maria Luisa Indiana, Elisabetta Sagone, Salvatore Luciano Orazio Fichera

**Affiliations:** Department of Educational Sciences, University of Catania, 95124 Catania, Italy; esagone@unict.it (E.S.); salvo.fichi@libero.it (S.L.O.F.)

**Keywords:** blindness, coping, cross-sectional study, deafness, parents, well-being

## Abstract

The main purpose of this cross-sectional study, carried out with deaf parents and blind parents, is to analyze the association of coping strategies, life satisfaction, well-being, and generalized self-efficacy, compared to a group of parents without a sensory loss. The Coping Orientation to Problems Experienced, Satisfaction with Life, Generalized Self-efficacy, and Psychological Well-Being scales were applied. Results indicate that: (1) deaf parents and blind parents search for social support, use avoidance, and turn to religion more than those without a sensory loss; (2) deaf parents are more satisfied with life than blind parents and those without a sensory loss; (3) deaf parents and blind parents perceive themselves as less efficacious than those without a sensory loss; (4) deaf parents and blind parents report lower psychological well-being (autonomy and personal growth) than those without a sensory loss, except for self-acceptance. Searching for social support and turning to religion are negatively associated with life satisfaction in deaf parents and those without a sensory loss; further, these coping strategies (together with avoidance) affect the psychological well-being of deaf parents and parents without a sensory loss. Future research could investigate deeper into the effects of these dimensions on well-being and the styles of parenting in these families.

## 1. Introduction

The majority of the studies consulted in scientific literature examined the relationships between life satisfaction, parental self-efficacy, well-being, and coping strategies predominantly in the parents of children with sensory impairments. Widespread literature reports that protective and risk factors positively or negatively influence the quality of life in families with deaf or blind children, deaf-blind children, or children with other developmental disabilities [1,2,3,4,5,6,7,8,9,10]. Among the protective factors, several scholars assume the coexistence of psychological and situational variables: parental resilience, optimism, hope orientation, adaptability and meaning-focused coping with stress, good communication skills with sensory disabled children, marital satisfaction, and social support from formal and informal networks [11,12,13,14,15]. On the other hand, among the risk factors, reduced labor possibilities, low levels of self-esteem, and high anxiety are predictors of low quality of life in families with children with sensory impairments or developmental disabilities [16,17]. It is noteworthy that the families of children with disabilities often go through critical events that can produce negative effects on the psychological well-being of each member and, in particular, of the parents [18,19,20,21,22,23,24]. The impact of deafness and blindness on family quality of life has been verified in various aspects of life, with reference to parental stress and high levels of anxiety [2,16,24,25,26,27,28], a sense of coherence [4,29], family interactions and social support [30,31], social relationships [32], and coping strategies [5,33,34,35]. Various studies indicated findings that are not univocal with each other. For example, it has been demonstrated that high anxiety is correlated to low psychological well-being in Spanish parents of blind children [36]; the cognitive coping style and psychological well-being in Iranian mothers of deaf children are significantly lower than those in mothers of children without a sensory loss [37]. As reported by Samadi et al. [6] for two groups of Iranian parents (121 parents of children with a diagnosis of autism spectrum disorders and 115 parents of children with an intellectual disability), mothers expressed significantly reduced emotional well-being (measured by the General Health Questionnaire [38]) compared to fathers. Furthermore, mothers and fathers of children with autism spectrum disorders report high parental stress (using the PSI-SF [39]). Concerning coping strategies, several studies regarding the role and the characteristics of parents with different conditions of disability to their own children provided relevant suggestions on the relationships between coping with stress and well-being (for children with autism: [40,41,42,43]; for children with developmental disability: [44]; for children with Down syndrome: [45]). To cite, Benson [41] observed that the use of an avoidant coping strategy by mothers of children with autism is associated with high levels of depression and anger, while the strategy of cognitive reframing is associated with high levels of well-being. Glidden et al. [44] found that mothers and fathers of children with developmental disabilities use more problem-focused than emotion-focused strategies; further, mothers of children with developmental disabilities, who report high levels of positive reappraisal, show high levels of subjective well-being, whereas those who obtain high levels of escape-avoidance display low levels of subjective well-being.

In addition, Sola-Carmona et al. [17] found that higher levels of material well-being, job satisfaction (valued with the two subscales of the Escala de Bienestar Psicológico [46]), and family satisfaction (measured using the ESFA-Escala de Satisfacción Familiar por Adjetivos [47]) are associated with lower levels of state-anxiety (measured with the State-Trait Anxiety Inventory-STAI) in a sample of Spanish parents of 61 blind children. Furthermore, in another study, realized by Sola-Carmona and colleagues [17], parents of visually impaired children who stated that their labor possibilities had been affected by having a disabled child, show high levels of anxiety. The high anxiety of these parents is caused by the progression of their child’s visual impairment, as well as poor and inadequate knowledge about their child’s disability. Parents who affirmed that having a blind child affected their work possibilities display lower self-esteem than those who state that their work possibilities are not affected. Following an opposite trend compared to previous findings, Kasin and colleagues [10] noted that the mental well-being of a group of American parents of deaf children is very similar to that of a non-clinical sample, while the sense of generalized self-efficacy and quality of life of parents of deaf children are higher. Moreover, Iranian mothers of children with a physical-motor disability reported lower levels of distress tolerance, and the use of problem-solving, physical control, social support, emotional monitoring, and cognitive evaluation (coping styles measured with the Coping Responses Inventory) than mothers of children without a disability [8]. Recently, Kurowska et al. [48] found that the parents of children with disabilities and parents of children with typical development predominantly use the active coping strategy, and their self-efficacy is associated with actively coping with stress.

### 1.1. Coping Strategies and Self-Efficacy in Parents of Sensory Disabled Children

The adaptation of families with deaf children or blind children to the various life challenges (inclusion in social context, maintaining supportive networks, and providing effective responses to the special needs of children: see Zanobini et al., in Italy [49]) is strongly influenced by the usefulness of parental resources in managing these stressors, increasing a sense of satisfaction with life, and influencing subjective well-being [17,35,36,50,51]. Among the parents, the exercise of functional coping strategies to overcome the adversities linked to sensory loss has positive effects on the reduction of parental stress [28], and the increase of self-efficacy in caregiving [52,53,54,55,56]. In a recent study by Gyllén, Magnusson, and Forsberg [57], concerning the sense of self-efficacy expressed by parents of children with congenital cataract, the results show that self-efficacy is viewed as the ability to find a balance between uncertainty and the acceptance of the condition of their children. Research underlines that high social support, if guaranteed by internal and external networks, is connected to high satisfaction with life and low stress in parents of disabled children [2,3,31,58,59]. Using a qualitative design of research, Shackelford [53] found that blind mothers are believed to successfully interact with their children and use multiple strategies to respond sensitively to their children’s signals. In addition, Rosenblum, Hong, and Harris [60] noticed that 67 parents with visual impairments state to utilize adequate strategies for their children’s safety, reporting the positive aspects of being a parent with visual impairment, using all methods of public transportation and assistive technology devices for homework, and providing information about the emotional impact on their children and others’ reactions to them as parents.

Research concerning the families of children with a sensory loss has shifted the attention from the theme of “family stress” to the consequences of adaptation, and the functional use of coping strategies to go beyond the difficulties linked to disability. This changing includes the connection among the main perspectives referred to adjustment, coping with stress, resilience, and quality of life [61,62,63,64,65,66]. Taking into consideration this last point of view, the principal models of coping chosen as theoretical frameworks are represented by the Folkman and Lazarus’ Ways of Coping [67] and the Carver, Scheier, and Weintraub’s model [68]. These authors indicated the existence of the following coping strategies: (1) orientation to problem-solving, consisting of actively coping with stressors and the suppression of competing and distracting activities; (2) positive reinterpretation, considered as an appropriate opportunity to act, transforming a stressful event into a potentially positive fact; (3) searching for social support, composed of seeking advice, searching for assistance or information, and receiving moral support; (4) avoidance, consisting of reducing one’s effort to deal with stressors, denying the presence of stress, and distracting from the problem; lastly, (5) turning to religion and absence of humor, referring to the trust in religious beliefs and the avoidance of humor to cope with critical situations. According to these models, an orientation to problem-solving and positive interpretation are active and functional coping strategies, while avoidance and turning to religious beliefs/absence of humor are passive and dysfunctional coping strategies. Additionally, Billings and Moos [69] specified that active coping is linked to cognitive and behavioral attempts to deal directly with the problem and its consequences, while passive coping is referred to the cognitive attempts to avoid confronting problems and behaviors aimed at indirectly reducing the emotional tensions. Passive coping strategies are often adopted by individuals when they are prone to decide that the circumstances cannot be altered or modified, and they need to accept the given situation as it is [70].

For the strategy of searching for social support, it is necessary to consider the different, and still fervent debate, on the issue of its functional or dysfunctional nature: almost all previously cited studies underlined that social support is a very useful factor for the promotion of well-being in parents who deal with the disability of children [9,71,72]. Therefore, when individuals receive social support from others, they are likely to utilize this aid to overcome their difficulties and to reach a psychological condition of emotional stability and subjective well-being. As found by Kyzar and colleagues [71], quality of life for families of children with deaf-blindness is partially explained by satisfaction with friends and family support networks and child-care services. However, the issue related to the idea that “providing social support may be more beneficial than receiving it” has been discussed in scientific literature in view of different aspects of social contact [73,74,75]. Regarding this idea, Lu and Argyle [73] noted that depending on other people to be supported can cause high levels of guilt and anxiety; similarly, Brown et al. [75] discovered that “feeling like a burden to others who presumably provide support is associated with increased suicidal tendencies” (p. 320). Therefore, when individuals are engaged in the searching of support from the others, they exclude the opportunity to personally deal with the problem and, consequently, to use the mandate as coping strategy to manage it. On the contrary, when individuals are involved in giving social support to others, they benefit from this behavior reducing their levels of stress and improving their conditions of health, and psychological well-being (in terms of purpose in life, sense of meaning and belonging) [76,77,78]. As recently found by Hemati Alamdarloo et al. [72], mothers of children with hearing impairments and mothers of children with visual impairments, similarly display lower perceived social support from family, friends and significant others than mothers of typically developed children. For these explanations, searching for social support can be viewed as a dysfunctional coping strategy for all individuals and, in particular, for parents of deaf or blind children.

Some scholars analyzed the coping strategies in parents of disabled children to ensure a condition of health for themselves and their children. Generally, parents who made extensive use of coping strategies focused on emotions are less satisfied with their life, report higher stress, and dissatisfaction with their partner, than parents who use coping strategies based on problem-solving [79,80,81]. Other studies proposed interesting results regarding the coping strategies adopted by parents of children with hearing impairments: Ishtiaq, Mumtaz, and Saqulain [82], and previously Bawalsah [83], found that the problem-focused engagement is more used than emotion-focused engagement. Additionally, Daud and colleagues [84] highlighted that religion, active coping, and acceptance are highly used by parents of children with hearing impairments, and the mothers tend to adopt religion, seeking emotional and instrumental support significantly more than the fathers do. These coping strategies have been seldom been investigated directly in relation to all components of well-being [22].

### 1.2. Psychological Well-Being and Life Satisfaction in Parents of Disabled Children

According to the eudaimonic perspective [85,86,87], the core dimensions of psychological well-being are constituted by self-acceptance, autonomy, personal growth, environmental mastery, positive relations with others, and purpose in life. These dimensions are useful to reach “optimal human functioning” and are represented by the ability to have a realistic perception of the self, including both good and bad qualities, together with the ability to make one’s own decisions without waiting for the approval of other people and to continuously grow and develop as a person, working for optimizing one’s full potential, having a sense of directedness, and intentionality changing life goals. Optimal human functioning is also linked to the ability to manage the environment which aligns with one’s values, or to choose the adequate environment accessible to an individual’s needs, together with the ability to develop caring relationships with others. Recently, research included in the construct of well-being and its subjective component (the satisfaction with life), integrated the components of hedonic (SWB) and eudaimonic well-being (PWB) into a comprehensive model of flourishing mental health [88,89,90,91] to explain positive effects on the quality of life of people [92]. For example, Ring and colleagues [92] found that individuals with high SWB/high PWB, report significantly higher scores in their quality of life’s measure than those with low SWB/low PWB.

Very little evidence has been noted regarding the relationships between life satisfaction, psychological well-being, and coping strategies, realizing comparisons among deaf parents, blind parents, and parents without sensory loss [71,93]. Some studies indicated that high well-being in families with children with atypical sensory development allowed families to become resilient [94,95,96]. Deaf parents and blind parents are highly able of care giving and parents with visual and physical limitations develop special ways to cope with their children [55,97]. In a qualitative study, Moghadam et al. [55] found that blind mothers adopt a close-mothering approach in caring for their children and this approach helps them to cope with their sensory limitations and decreases their level of child-related anxiety. Further, deaf children of deaf parents perform academically [98], linguistically [99,100,101], and socially [101,102] better than deaf children of parents without a sensory loss. Furthermore, Pruteanu and Sandovici [103] found that the positive affective disposition and satisfaction with life expressed by parents of children with total hearing impairments are positively associated with functional cognitive strategies involved in regulating emotions in negative life events, as well as dispositional optimism and high resilience. These findings are generally explained with high levels of coping and adequate development of substitute skills that are useful to provide care for children.

Given the importance of parental well-being in the care of children (Pinquart and Pfeiffer’s meta-analysis [104]), a better understanding of the impact of deafness and blindness on the family lifecycle and members’ perceptions of quality of life is noteworthy.

## 2. Purpose of Study and Hypotheses

The principal aim of this study is to examine the association of coping strategies with psychological well-being, self-efficacy, and satisfaction with life expressed by deaf parents and blind parents, compared to a group of parents without a sensory loss. The choice of participants in their role of caregivers (mothers and fathers of children with sensory impairments) is linked to a broad project concerning the analysis of creativity and well-being of children with visual impairments developed at Department of Educational Sciences, University of Catania (Sicily, Italy).

Considering the abovementioned, and not often coherent, literature about the role of deafness and blindness in family quality of life, we hypothesize the following results:

**Hypothesis** **1** **(H1).** *Deaf parents and blind parents use more dysfunctional coping strategies (avoidance, turning to religion, and searching for social support) than parents without a sensory loss.*

**Hypothesis** **2** **(H2).** *Deaf parents and blind parents report lower life satisfaction than parents without a sensory loss.*

**Hypothesis** **3** **(H3).** *Deaf parents and blind parents show lower self-efficacy than parents without a sensory loss.*

**Hypothesis** **4** **(H4).** *Deaf parents and blind parents express lower psychological well-being than parents without a sensory loss.*

Consistently with the relevance of coping strategies for generalized self-efficacy, life satisfaction, and psychological well-being, we expect that:

**Hypothesis** **5a** **(H5a).** *Adaptive coping strategies (orientation to problem-solving and positive interpretation) are positively associated with life satisfaction expressed by deaf parents, blind parents, and parents without a sensory loss: this means that the more these parents use coping functional strategies to adapt to stressful events, the more they are satisfied with their everyday life;*

**Hypothesis** **5b** **(H5b).** *Adaptive coping strategies (orientation to problem-solving and positive interpretation) are positively associated with the generalized self-efficacy expressed by deaf parents, blind parents, and parents without a sensory loss: this means that the more these parents use helpful coping strategies to overcome critical situations, the more they perceive themselves as efficacious;*

**Hypothesis** **5c** **(H5c).** *Adaptive coping strategies (orientation to problem-solving and positive interpretation) are positively associated with the psychological well-being of deaf parents, blind parents, and parents without a sensory loss: therefore, the more these parents use positive coping strategies, the more they display high levels in the dimensions of psychological well-being.*

Consistent with the previous assumptions, we believe that dysfunctional coping strategies (avoidance, turning to religion, and searching for social support) are negatively associated with life satisfaction, generalized self-efficacy, and psychological well-being in deaf parents, blind parents, and parents without a sensory loss: therefore, the more these parents use dysfunctional coping strategies, the less they are satisfied with their life, express low levels of self-efficacy, and report a condition of reduced psychological well-being.

**Figure 1:**
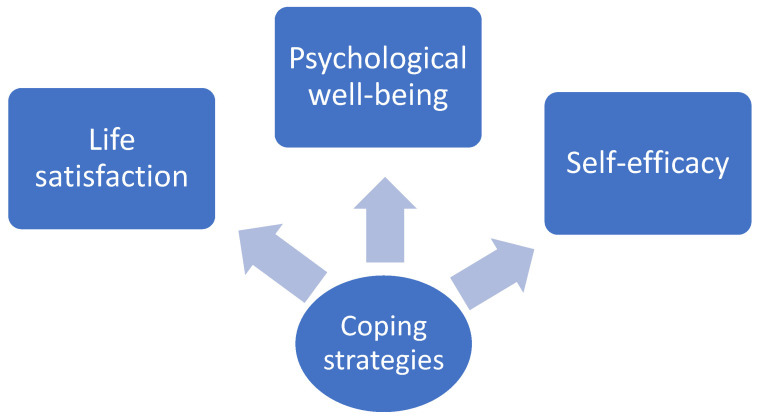
Research design.

The research design is reported below (Figure 1), with coping strategies as an independent variable and life satisfaction, psychological well-being, and self-efficacy as dependent variables.

## 3. Method and Measures

### 3.1. Sample

The sample consists of 101 Sicilian parents, aged between 30 and 54 years, divided into 34 deaf parents (20 mothers and 14 fathers), 31 blind parents (17 mothers and 14 fathers), and 36 parents without a sensory loss (15 mothers and 21 fathers). The mean age of deaf parents (*M*_age_ = 39.9, *sd* = 5.9) and of blind parents (*M*_age_ = 40.1, *sd* = 5.7) is comparable to that of parents without a sensory loss (*M*_age_ = 41.3, *sd* = 4.9). The age of children ranges from 6 to 12 years in all families. The detailed information about gender and average age of children was not collected by the authors. No information about the socio-economic status of parents has been included in this analysis due to the missing responses in a lot of cases. The majority of deaf parents (30 out of 34 deaf parents) use Italian Sign Language to communicate with members of their family and others; the remaining deaf parents utilize other communication systems (such as oral speech). Blind parents adopted screen readers, Braille displays, and speech recognition software. All parents have been invited to participate in this study following the clarifications provided by researchers and their participation was voluntary.

The study inclusion criteria are as follows: (1) adults < 60 years of age, (2) married parents, (3) biological fathers and mothers of children, (4) previously diagnosed bilateral hearing loss (congenital hearing loss) and absence of cochlear implants or hearing aids, (5) officially diagnosed blind adults (congenital blindness), (6) the use of Braille coding for blind parents and the use of Italian Sign Language for deaf parents, and (7) absence of additional disabilities in the parents with a sensory deficit. For both groups of parents, the presence of dual sensory loss or multi-sensory impairments, and the inefficient reading comprehension previously verified through the instructions given by the authors, were chosen as exclusion criteria.

Formal consent has been obtained prior to starting the study using a written cover letter to explain the aim of this investigation. The study started only after further clarification was provided by researchers. The authors respected the Ethical Code for Italian psychologists (L. 18.02.1989, n.56), the Legislative Decree for the privacy of provided data (DLGS 196/2003), and the Ethical Code for Psychological Research (27 March 2015) established by the Italian Psychologists Association.

### 3.2. Measures

A structured questionnaire has been used to analyze the principal constructs of this study, including the Life Satisfaction Scale (LSS [105,106]), the Generalized Self-Efficacy Scale (GSEs [107]), the 18-item version of the Psychological Well-being Scales (PWBs [108,109]), and the Coping Orientation to Problems Experienced (COPE [68,110]).

The Italian version of the Satisfaction with Life Scale [106] was chosen to measure the subjective satisfaction and consists of five items (e.g., “In most of my ways my life is close to my ideal”) with responses on a 7-point Likert scale (1 = strongly disagree, 7 = strongly agree). The internal consistency of LSS for this study was good with value of α equal to 0.86).

The Generalized Self-Efficacy Scale [107] was used to assess the general sense of perceived self-efficacy to predict coping with daily hassles, as well as adaptation after experiencing all types of stressful life events. This scale is composed of 10 items (“When I am confronted with a problem, I can usually find several solutions”, “I can solve most problems if I invest the necessary effort”, “If someone opposes me, I can find the means and ways to get what I want”) on a 4-point Likert scale ranging from 1 (corresponding to “not at all true”) to 4 intervals (corresponding to “exactly true”). Total score ranges from 10 to 40 points. For this scale, the internal consistency was satisfactory for our sample with value of α equal to 0.82.

The Psychological Well-being Scales [108] were used as a self-report inventory with a set of items for each of which parents evaluate themselves on a 6-point Likert scale, with 1 corresponding to “strong disagreement” and 6 corresponding to “strong agreement”. We applied the Italian short form version of the PWBs with 18 items [109] grouped in the following six subscales: (1) autonomy (e.g., “I tend to be influenced by people with strong opinions”, reverse item); (2) environmental mastery (e.g., “In general, I feel I am in charge of the situation in which I live”); (3) purpose in life (e.g., “I live life one day at a time and do not really concern myself with things about the future”); (4) positive relations with others (e.g., “I have not experienced many warm and trusting relationships with others”, reverse item); (5) personal growth (e.g., “For me life has been a continuous process of learning, changing, and growth”); and (6) self-acceptance (e.g., “When I look at the story of my life, I am pleased with how things have turned out”). Responses are computed for each subscale (about half of the responses are reverse scored) and high scores indicate that the respondents report high levels, in a given dimension, in their own life. The internal consistency of PWBs for this study is acceptable, with value of α equal to 0.74.

The Coping Orientation to Problems Experienced is a measure of the extent in which individuals use skills and strategies to cope with stressful events [110]. It is composed of 60 sentences valued by parents with the following choices from 1 to 4 points: “I usually do not do this at all”, “I usually do this a little bit”, “I usually do this a medium amount”, and “I usually do this a lot”. The coping strategies included in this measure have been analyzed using the Carver et al.’s coping model [69]. The items are grouped into five subscales (according to the New Italian version created by Sica et al. [110]): (1) searching for social support, (2) avoidance, (3) positive reinterpretation, (4) orientation to problem solving, and (5) turning to religion. In detail, the strategy of searching for social support includes the seeking of social support both for instrumental and emotional/moral reasons; the strategy of avoidance uses the denial of stress, behavioral and mental disengagement, with the reduction of one’s effort to deal with stressors and distraction from thinking about the problem; the strategy of positive reinterpretation is referred to as the attitude of waiting until an appropriate opportunity to act presents itself (e.g., restrain coping) and construing a stressful event in positive terms (e.g., positive reinterpretation and growth); the strategy of orientation to problem solving consists of taking active steps to circumvent the stressor (e.g., active coping), thinking about how to cope with stressors (e.g., planning), and continuing to be distracted by other things (corresponding to the suppression of competing activities); lastly, the strategy of turning to religion/absence of humor is referred to having a trust in religion and avoiding the use of humor to cope with critical situations. The internal consistency of COPE for this study was very good (α = 0.90).

### 3.3. Procedures

After receiving approval for this study by the Institutional Ethic Review Board of Psychology Research, the chosen scales have been individually administered in the presence of, and with the aid of, the translation realized by the Italian LIS interpreter for deaf parents to make highly comprehensible the statements. The LIS interpreter is a specialized and certified expert who translates from Italian to the Italian Sign Language and was chosen from the Centers for Deafness sited in Catania and the provinces. The translation was performed live for each deaf parent and contemporarily to the presentation by the researchers. Each statement has been simplified according to the structural properties of “A Grammar of LIS” [111].

For blind parents, the adaptation of scales in Braille coding has been previously created by an expert in Software for Braille Translation (WinBraille for Windows, Italy). The use of this assistive technology device can improve the autonomy, quality of life, and social inclusion of individuals with visual impairments [112].

For parents without sensory loss, no precaution or expedient for administration of the questionnaire has been required.

Data have been collected before the first general lockdown for the COVID-19 outbreak, complying with the rules of social distancing already existing in the North of Italy and established by the Italian Government, while data analysis was carried out after the second lockdown.

### 3.4. Data Analysis

Statistical analysis was carried out with the IBM SPSS 20, using analysis of variance (ANOVA) and linear regressions. Each group of parents has been used as independent variable, while mean scores obtained in life satisfaction, psychological well-being, generalized self-efficacy, and coping strategies, have been used as dependent variables. The analysis of ANOVA with Bonferroni’s post-hoc test has been carried out to verify the significant differences among the group of parents. Even if the role differences in this type of research is noteworthy [5,6,22], we have decided to exclude from these analyses two important variables: age and role of parents (mothers and fathers), due to the imbalance between the number of mothers and that of fathers in this convenient sample; and the omission of information regarding the age of several parents in data collection procedure. Furthermore, the analysis of linear regressions has been applied for measuring the associations between coping strategies and well-being, generalized self-efficacy, and life satisfaction both for deaf parents and blind parents.

## 4. Results

### 4.1. Descriptive Analyses for Coping Strategies

Considering the H1, results indicate that deaf parents mainly use positive reinterpretation (*M* = 37.91, *sd* = 4.5), searching for social support (*M* = 33.05, *sd* = 6.7), and problem-solving orientations (*M* = 32.52, *sd* = 4.2); additionally, blind parents tend to adopt positive reinterpretation (*M* = 38.29, *sd* = 4.5), searching for social support (*M* = 33.19, *sd* = 6.9), and problem-solving orientations (*M* = 32.77, *sd* = 4.2); finally, parents without a sensory loss predominantly use positive reinterpretation (*M* = 37.30, *sd* = 6.9) and problem-solving orientations (*M* = 34.36, *sd* = 6.4). As shown in Table 1, relevant differences in coping strategies are observed for the group of parents: using the ANOVA, deaf parents and blind parents tend to adopt searching for social support (for *p* < 0.001), avoidance (for *p* < 0.001), and the turning to religion strategy (for *p* = 0.015) more than parents without a sensory loss. Bonferroni’s post-hoc test corroborates significant differences in relation to searching for social support between deaf parents and those without a sensory loss (*p* < 0.001) and between blind parents and those without a sensory loss (*p* < 0.001), as well as in relation to avoidance (both for *p* < 0.001). A similar datum was observed for turning to religion between deaf parents and those without a sensory loss (*p* = 0.04) and between blind parents and those without a sensory loss (*p* = 0.032).

### 4.2. Descriptive Analyses for Satisfaction with Life

In relation to H2, statistical analysis shows that deaf parents are more satisfied with their everyday life (*M* = 27.61, *sd* = 3.4) than blind parents (*M* = 23.19, *sd* = 3.4) and those without a sensory loss (*M* = 21.08, *sd* = 9.4) (*F*_(2,98)_ = 9.721, *p* < 0.001). Bonferroni’s post-hoc test indicates a significant difference both between deaf parents and those without a sensory loss (*p* < 0.001) and between deaf parents and blind parents (*p* = 0.01), confirming the obtained results in this dimension.

### 4.3. Descriptive Analyses for Generalized Self-Efficacy

In relation to H3, results demonstrate that deaf parents (*M* = 30.5, *sd* = 4.2) and blind parents (*M* = 29.9, *sd* = 4.3) perceive themselves as less efficacious than parents without a sensory loss (*M* = 32.83, *sd* = 4.7) (*F*_(2,98)_ = 4.147, *p* = 0.019). Bonferroni’s test confirms a significant difference only between blind parents and parents without a sensory loss (*p* = 0.026).

### 4.4. Descriptive Analyses for Psychological Well-Being

For H4, descriptive analysis indicates that deaf parents reach high mean scores of environmental mastery (*M* = 14.23, *sd* = 1.6), followed by self-acceptance (*M* = 13.61, *sd* = 1.7) and personal growth (*M* = 13.17, *sd* = 1.05); blind parents obtain high scores of environmental mastery (*M* = 14.29, *sd* = 1.6), self-acceptance (*M* = 13.54, *sd* = 1.7), and personal growth (*M* = 13.19, *sd* = 1.07); finally, parents without a sensory loss report high scores of personal growth (*M* = 14.66, *sd* = 2.6), environmental mastery (*M* = 13.91, *sd* = 2.1), and autonomy (*M* = 13.13, *sd* = 2.6).

Partial differences for each group of parents in these dimensions were observed (Table 2). Excluding environmental mastery and purpose in life, deaf parents and blind parents report lower levels of psychological well-being than parents without a sensory loss (*F*_(2,98)_ = 4.552, *p* = 0.013); only for self-acceptance, do deaf parents and blind parents show higher levels than parents without a sensory loss (*F*_(2,98)_ = 3.826, *p* = 0.025). Bonferroni’s post-hoc test confirms significant differences in autonomy and personal growth between deaf parents and parents without a sensory loss (autonomy: *p* = 0.030; personal growth: *p* = 0.002), as well as between blind parents and parents without a sensory loss (autonomy: *p* = 0.013; personal growth: *p* = 0.004).

### 4.5. Associations between Coping Strategies and Life Satisfaction, Self-Efficacy, and Well-Being

To analyze the associations among coping strategies used by all parents regarding life satisfaction, generalized self-efficacy, and psychological well-being (H5a, H5b and H5c), we applied linear regressions (separately for each group of parents) using the COPE as an independent variable, whereas LSS, GSEs, and PWBs were dependent variables. As expected by H5a, some coping strategies are partially related to life satisfaction. In detail, searching for social support is negatively associated with life satisfaction only for deaf parents (*β* = −0.652, *t* = −2.932, *p = 0*.007) and turning to religion is positively related to life satisfaction only for parents without a sensory loss (*β* = 0.497, *t* = 2.951, *p* = 0.006). Regarding to H5b, results indicated that coping strategies do not affect self-efficacy in all three groups of parents.

As hypothesized in H5c, some coping strategies are associated with psychological well-being and these results are clear for both deaf parents and blind parents:

- Searching for social support is negatively associated with autonomy, both for deaf parents (*β* = −0.957, *t* = −5.418, *p* < 0.001) and blind parents (*β* = −1.00, *t* = −4.478, *p* < 0.001), but positively concerning relations with others (*β* = 0.304, *t* = 2.075, *p* = 0.04) for parents without a sensory loss;
- Avoidance is negatively associated with environmental mastery (*β* = −0.465, *t* = −3.098, *p* = 0.004), relations with others (*β* = −0.696, *t* = −4.884, *p* < 0.001), personal growth (*β* = −0.499, *t* = −3.574, *p* = 0.001) and self-acceptance (*β* = −0.429, *t* = −3.122, *p* = 0.004) for parents without a sensory loss. Furthermore, avoidance is negatively associated with personal growth for both deaf parents (*β* = −0.626, *t* = −2.601, *p* = 0.015) and blind parents (*β* = −0.736, *t* = −2.843, *p* = 0.009);
- Positive reinterpretation is positively associated with personal growth (*β* = 0.652, *t* = 2.369, *p* = 0.025) and negatively with self-acceptance (*β* = −0.699, *t* = −3.200, *p* = 0.003) for deaf parents;
- Turning to religion is positively related to personal growth (*β* = 0.619, *t* = 3.990, *p* < 0.001) and self-acceptance (*β* = 0.800, *t* = 5.228, *p* < 0.001) for parents without a sensory loss.

## 5. Discussion

The analysis of the association between coping strategies and satisfaction with life, generalized self-efficacy, and well-being in deaf and blind parents represents the main purpose of this cross-sectional study. Substantially, results confirmed the general hypotheses, excluding life satisfaction. According to H1, deaf parents and blind parents use more dysfunctional coping strategies than parents without a sensory loss. In this perspective, the searching for social support and turning to religious beliefs are viewed as a synonym of mandate to other people, reducing one’s own personal action or contribution for overcoming critical situations. Only in reference to blind parents, these results are in contrast with those revealed by Shackelford [53] and Rosenblum et al. [60], according to which parents with sensory disabilities are prone to adopt positive and useful coping strategies to manage their own disability and the disability of their children. Referring to deaf parents, we did not find any findings about the coping strategies mainly used by this group or predominantly adopted comparing sensory disabled parents with those without a sensory deficit, but only data collected from the parents of deaf or blind children.

Contrary to H2, the results indicate that life satisfaction is higher in deaf parents compared with the other parents. With reference to this construct, as previously found by Sagone [22] in the same socio-cultural Italian context, satisfaction with life is more highly reached by deaf parents compared to parents without sensory disabilities. This result is scarcely confirmed by other studies carried out by other researchers. From the consulting of scientific literature concerning this issue, also in this case, we did not find any noteworthy results about the levels of life satisfaction experienced by these groups of sensory disabled parents. It is possible to cite the empirical evidence recently observed by Sola-Carmona with reference to the two components of life satisfaction and job and family satisfaction, in parents of blind children [17]: the more these parents (predominantly, mothers) experience high levels of satisfaction as a core dimension of well-being, the more they show low anxiety. Additionally, Pruteanu and Sandovici [103] found that parents of children with total hearing impairments show significant correlations between positive affective disposition and satisfaction with life, even if these results are expressed by parents without sensory disability.

Consistently with H3, deaf parents and blind parents perceive themselves as less efficacious than parents without a sensory loss, reporting low levels of generalized self-efficacy. Little is known about the in-depth meaning of parental self-efficacy and self-esteem (two important components of self-concept) in this population, while several findings derive from the studies were realized from parents of children with visual and auditory deficits. For example, as reported by Kasin et al. [10], parents of deaf children score higher in generalized self-efficacy and quality of life than parents of children with a typical sensory development. Additionally, as reported by Gyllén and colleagues [57], the self-efficacy of parents of children with congenital cataracts is considered as the ability to reach an equilibrium between the sense of uncertainty and the acceptance of their child’s condition.

Lastly, for H4, deaf parents and blind parents report lower psychological well-being (autonomy and sense of personal growth) than parents without a sensory loss; for the group of blind parents, this result can be viewed as consistent with that obtained by Pinquart and Pfeiffer’s meta-analysis [104], according to which, adults with visual impairments show lower psychological well-being than sighted adults. Only one result differs from the previous evidence: deaf parents and blind parents reach higher levels of self-acceptance than parent without a sensory loss. This last datum could be explained by the idea that these parents learn to accept their sensory atypical condition and to adequately balance out this lack or deprivation [99]; for this reason, this fact would be related to higher life satisfaction (mainly for deaf parents). The majority of studies referring to well-being are carried out with parents of children with visual impairments, sensory deficits, or postlingual deafness [10,93]. Accordingly, Kasin and colleagues [10] found that scores of well-being in parents of deaf children are very similar to those obtained by parents of children with typical sensory development.

Considering the associations of coping strategies assumed by all parents of this study with life satisfaction (H5a), generalized self-efficacy (H5b), and psychological well-being (H5c), deaf parents who utilize dysfunctional coping strategies (mainly, searching for social support) are less satisfied with their everyday life. On the contrary, the parents without a sensory loss who adopt the coping strategy of turning to religious beliefs are more satisfied than the others. Furthermore, for the association between coping strategies and psychological well-being, it is partially confirmed: the deaf parents and blind parents who make use of maladaptive coping strategies (avoidance and searching for social support) display low psychological well-being and, specifically, a reduced sense of personal growth and autonomy. These data can be explained by considering that avoidance and searching for social support are viewed as dysfunctional coping strategies [73,74,75]. The well-being of deaf parents and blind parents can be threatened and put at risk by behaviors and personal attitudes such as denying the disability of children. However, these results were also observed in parents without a sensory loss: avoidance negatively affects environmental mastery, relations with others, a sense of personal growth, and self-acceptance, whereas turning to religion positively affects personal growth and acceptance of self. Concerning the relationships between coping strategies with stress and well-being in parents of autistic children [40,41,42,43], parents of children with developmental disabilities [44] and parents of children with Down syndrome [45], it has been observed that the mothers of children with autism, who use the avoidant coping strategy, display high levels of depression and anger, while those who adopt the cognitive reframing reach high levels of well-being [41]. Additionally, mothers and fathers of children with developmental disabilities are more likely to choose more problem-focused than emotion-focused strategies. Furthermore, mothers who use positive reappraisal display high subjective well-being, whereas those who adopt an escape avoidance strategy display low subjective well-being [44].

As indicated in the introduction, studies in this field of research did not provide any results concerning the influence of maladaptive and dysfunctional coping strategies in groups of deaf and/or blind parents. There is little research about this topic and, especially, with reference to parents with sensory disabilities. The present study is the second analysis referring to coping strategies that involved parents with disabilities (deafness or blindness) in an Italian context. It can be considered as a starting point to understand the psychological condition of parents in the caregiving of children and their education skills. In a previous study realized in the same social context, Sagone [22] found that a group of Italian deaf parents used dysfunctional coping strategies (valued using the COPE) and reported lower levels of autonomy, personal growth, positive relations with the others, and purpose in life (measured with the Ryff’s Psychological Well-Being Scales) compared to the parents without sensory loss, although deaf parents were more satisfied with life than the others. Furthermore, searching for social support and avoidance negatively influenced the life satisfaction expressed by deaf parents and those without sensory loss, while turning to religion positively affected life satisfaction and psychological well-being of both parents.

## 6. Limitations and Future Research

The current study presents some weaknesses. The paucity of sources concerning the characteristics of these participants involved in this study (that is, deafness and blindness) made it very difficult to compare the current results with those of other studies, not only in an Italian context but also in other countries. Furthermore, the authors cannot consider this sample as being representative, considering the fact convenient sampling and the unbalanced number of mothers and fathers in each group are evident. Future research on styles of parenting, together with other dependent variables linked to well-being (e.g., resiliency and quality of life), will be required to better understand their relationships.

Another limitation of this study concerns the unique use of a structured questionnaire with close-ended questions; the authors believe that an in-depth interview could be a useful tool for examining the coping strategies and dimensions of well-being expressed by these parents according to the autobiographical qualitative approach.

Despite the limitations of the current study, its’ significant results recommend that additional research should be undertaken to a) replicate these findings with a larger sample, b) analyze the motivations underlying the tendency to use the searching of social support as a dysfunctional coping strategy, and c) estimate the direct effects of coping strategies on the psychological well-being of families with disabled parents.

## 7. Conclusions

The present study allows us to better understand the differences between the psychological dimensions included in the quality of life in parents who go through the direct experience of sensory disability. This point can be considered as a strength of this cross-sectional study in an Italian context. The findings of the current study derive from a “snapshot” of all groups of parents chosen for this comparative study before the pandemic, without reference to the psychological condition experienced after the COVID-19 outbreak. It would have been interesting to compare the scores obtained in each scale (LSs, GSEs, PWBs, and COPE) by these parents before and after the pandemic. We are aware that the variable “COVID-19 lockdown” can affect the use of coping strategies, life satisfaction, self-efficacy, and psychological well-being in the parents of deaf and/or blind children, with the consequent risks of marginalization and exclusion by social life. In addition, we agree with the idea that the obtained results can undergo some changes during and after the COVID-19 outbreak.

The use of a comparative group of parents without a sensory loss admits us to grasp the extent of similarities and differences in the psychological health (well-being and sense of self-efficacy) of parents with deaf or blind children. It can help professionals to address the further interventions on parental well-being and, overall, to rethink ideas according to which providing social support may be more beneficial than receiving it. Future analyses will allow us to test the role of other predictors (e.g., optimistic orientation toward the future) and protective factors (e.g., resilience and hope) in the well-being of these parents to better understand the complexity of the phenomenon of disability in family life span. As found in other studies, it is noteworthy to underline that optimism is a moderating factor between disability of children and the well-being of parents (especially, mothers) [113]; this psychological factor indirectly affects cognitive emotion regulation and perceived social support [114]. In addition, positive relationships are observed between hope and the well-being of parents with disabled children [115]. There is a deep need to develop individualized programs for family psychological support and parent training, as it would be useful to stimulate the use of constructive and adaptive coping strategies both with the disability of children and disability of their parents. The parent training programs support parents in managing issues that are common for families of disabled children: they permit parents to establish good family routines, manage family stress and increase educational practices of self-care, and to identify step-by-step the special needs of children. Furthermore, the training programs provide some of the best practices about parental skills regarding safety and childcare. These educational practices are included both in the guidelines for a successful parenting in at-risk conditions for developmental trajectories of disabled children, and face-to-face support groups focused on modeling and role-playing methods. Generally, these training courses are carried out in a face-to-face or online modality and involve mothers and fathers without disabilities; in this case, it would be useful to apply these training programs in families with disabled parents (sensory disability or intellectual delay).

## Figures and Tables

**Table 1:** Coping strategies: differences for groups of parents.

Variables of COPE	Type of Group	*M*	*sd*
Searching for social support	Group I	33.05	6.74
	Group II	26.44	6.49
	Group III	33.19	6.95
Avoidance	Group I	31.50	5.73
	Group II	24.72	6.61
	Group III	31.48	5.89
Positive reinterpretation	Group I	37.91	4.51
	Group II	37.30	6.90
	Group III	38.29	4.52
Orientation to problem solving	Group I	32.53	4.22
	Group II	34.36	6.43
	Group III	32.77	4.25
Turning to religion	Group I	21.71	4.49
	Group II	18.83	5.33
	Group III	21.90	4.52

Note: Group I: deaf parents; Group II: parents without sensory loss; Group III: blind parents.

**Table 2:** Dimensions of well-being: differences for groups of parents.

Variables of PWBs	Type of Group	*M*	*sd*
Autonomy	Group I	11.21	3.34
	Group II	13.14	2.62
	Group III	10.94	3.28
Environmental mastery	Group I	14.23	1.61
	Group II	13.92	2.11
	Group III	14.29	1.68
Personal growth	Group I	13.18	1.05
	Group II	14.67	2.66
	Group III	13.19	1.08
Relations with others	Group I	11.15	2.50
	Group II	12.75	3.34
	Group III	11.16	2.50
Purpose in life	Group I	9.32	2.01
	Group II	10.81	4.03
	Group III	9.42	2.04
Self-acceptance	Group I	13.62	5.69
	Group II	12.11	4.81
	Group III	13.55	5.90

Note: Group I: deaf parents; Group II: parents without sensory loss; Group III: blind parents.

## Data Availability

The data presented in this study are available on request from the corresponding author (M.L.I.).
